# Hederagenin from the leaves of ivy (Hedera helix L.) induces apoptosis in human LoVo colon cells through the mitochondrial pathway

**DOI:** 10.1186/1472-6882-14-412

**Published:** 2014-10-24

**Authors:** Bao-Xin-Zi Liu, Jin-Yong Zhou, Yu Li, Xi Zou, Jian Wu, Jun-Fei Gu, Jia-Rui Yuan, Bing-Jie Zhao, Liang Feng, Xiao-Bin Jia, Rui-Ping Wang

**Affiliations:** Department of Oncology, Jiangsu Province Hospital of Traditional Chinese Medicine, The Affiliated Hospital of Nanjing University of Chinese Medicine, Nanjing, 210029 P. R. China; Key Laboratory of New Drug Delivery Systems of Chinese Meteria Medica, Jiangsu Provincial Academy of Chinese Medicine, Jiangsu, Nanjing 210028 P. R. China

**Keywords:** Hederagenin, Apoptosi, Mitochondrial pathway, Colon cancer

## Abstract

**Background:**

Colorectal cancer has become one of the leading cause of cancer morbidity and mortality throughout world. Hederagenin, a derivative of oleanolic acid isolated from the leaves of ivy (Hedera helix L.), has been shown to have potential anti-tumor activity. The study was conducted to evaluate whether hederagenin could induce apoptosis of human colon cancer LoVo cells and explore the possible mechanism.

**Methods:**

MTT assay was used for evaluating cell viability while Annexin V-FITC/PI assay and Hoechst 33342 nuclear stainining were used for the determination of apoptosis and mitochondrial membrane potential. DCFH-DA fluorescence staining and flow cytometry were used to measure ROS generation. Real-time PCR and western blot analysis were performed for apoptosis-related protein expressions.

**Results:**

MTT assay showed that hederagenin could significantly inhibit the viability of LoVo cells in a concentration-dependent and time-dependent manner by IC_50_ of 1.39 μM at 24 h and 1.17 μM at 48 h. The apoptosis ratio was significantly increased to 32.46% and 81.78% by the induction of hederagenin (1 and 2 μM) in Annexin V-FITC/PI assay. Hederagenin could also induce the nuclear changes characteristic of apoptosis by Hoechst 33342 nuclear stainining under fluorescence microscopy. DCFH-DA fluorescence staining and flow cytometry showed that hederagenin could increase significantly ROS generation in LoVo cells. Real-time PCR showed that hederagenin induced the up-regulation of Bax and down-regulation of Bcl-2, Bcl-xL and Survivin. Western blotting analysis showed that hederagenin decreased the expressions of apoptosis-associated proteins Bcl-2, procaspase-9, procaspase-3, and polyADP- ribosepolymerase (PARP) were increased, while the expressions of Bax, caspase-3, caspase-9 were increased. However, there was no significant change on caspase-8.

**Conclusions:**

These results indicated that the disruption of mitochondrial membrane potential might contribute to the apoptosis of hederagenin in LoVo cells. Our findings suggested that hederagenin might be a promising therapeutic candidate for human colon cancer.

## Background

Mounting evidence showed that colorectal cancer is the most frequently diagnosed cancer and the second leading cause of cancer-related death [1, 2]. Several epidemiological studies have reported that the mean cumulative incidence rate of colorectal cancer is 3.1% at 10 years [3, 4]. The risk of this disease is multifactorial, such as age, environmental, genetic, and dietary factors. The etiology of colorectal cancer is also limited, Currently, therapeutic methods for human colorectal cancer include radiotherapy, chemotherapy and surgery [5]. However, these strategies for the treatment of colorectal cancer are not satisfactory. Therefore, effective strategy needs to be developed to decrease the morbidity and mortality of colorectal cancer.

Apoptosis plays an important role in the transition from normality to malignancy on colorectal cancer and the regulation of tissue development and homeostasis [6]. Apoptotic cell can be executed by the extrinsic and the intrinsic activated signaling pathways in the intestinal tract [7]. There was a growing body of evidence from mutiple studies showed the fact that the apoptosis was related closely to the loss of mitochondrial membrane integrity [8, 9]. The over-generation of reactive oxygen species (ROS) may contribute to the apoptosis through regulating Bcl family proteins, releasing cytochrome c from the mitochondria, and activating caspases such as caspase-3 and caspase-9 [9]. It is a promising emerging strategy for the prevention and treatment of colorectal cancer via inducing apoptosis which was mediated by mitochondria.

Hederagenin, a derivative of oleanolic acid isolated from the leaves of ivy (Hedera helix L.), has a strong anti-tumor effect both *in vitro* and *in vivo* [10]. Its saponin macranthoside B has been shown to induce apoptosis in various kinds of cancer cells through activating the caspase cascades for intrinsic pathways and regulating the protein level of Bcl-2 and Bax, as well as Bax/Bcl-2 ratio [11]. In addition, the bidesmosides of hederagenin are able to induce apoptosis in HeLa cells [12]. However, there is a limited report on the induction of hederagenin on apoptosis in colorectal cancer cells. In this study, we examined whether hederagenin could induce apoptosis in LoVo cells to inhibit the proliferation of tumor cells. Furthermore, the mechanism of hederagenin on inducing apoptosis of LoVo colon cells was also explored, including the mitochondrial pathway and the expressions of apoptosis-related proteins.

## Methods

### Reagent

**Figure 1:**
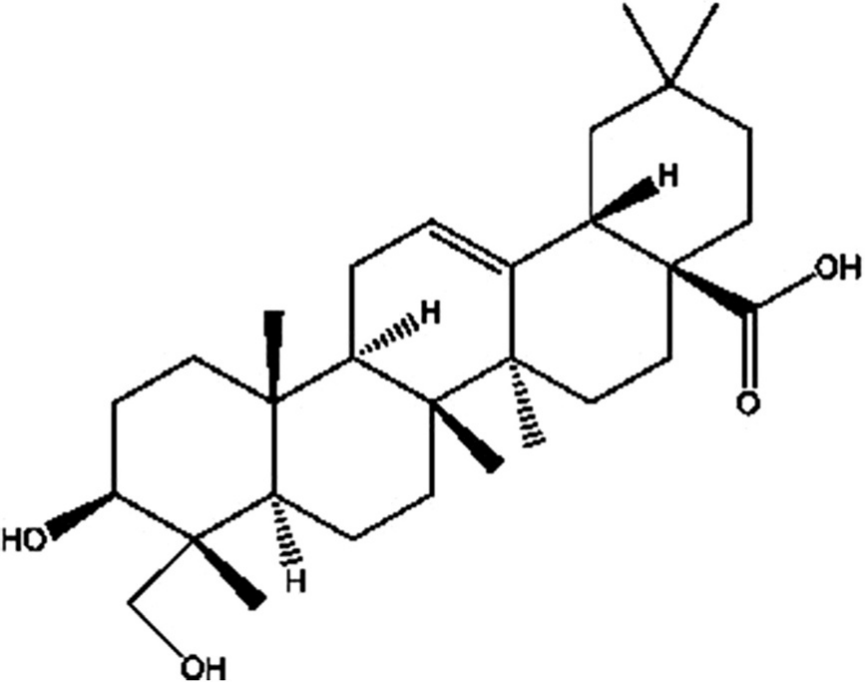
**The chemical structure of hederagenin (C**_**30**_ **H**_**48**_ **O**_**4**_ **, molecular weight = 472.70).**

Hederagenin ((3 beta, 4alpha)-3,23-dihydroxyolean-12-en-28-oic acid, C_30_H_48_O_4_, molecular weight = 472.70, purity ≥ 97%) was isolated in our laboratory from the leaves of ivy (Hedera helix L.) which was collected from Shaoyang City, Hunan Province of China at October 22, 2012. The voucher specimen of the leaves has been deposited at the Plant Resources Herbarium, Jiangsu Provincial Academy of Chinese Medicine (No. ZYY-2012103004). The chemical structure of hederagenin was shown in Figure 1. Dulbecco’s modified Eagle’s medium (DMEM) and bovine serum were from Life Technologies (Grand Island, NY). TRIzol reagents were obtained from Invitrogen (Carlsbad, CA). JC-1 detection kit was purchased from KeyGEN Biotechnology (Nanjing, China). Primescript RT reagent kit with gDNA Eraser and SYBR Premix Ex Taq were from TaKaRa (Dalian, China). Antibodies against caspase-3, caspase-9, caspase-8 were from Cell Signaling Technology (Beverly, MA). Antibodies against Bcl-2 or Bax were purchased from Santa Cruz Biotechnology (Santa Cruz, CA). Annexin V-fluorescein isothiocyanate (FITC) and propidium iodide (PI) apoptosis detection kit was from BD Biosciences (San Diego, CA). The horseradish peroxidase (HRP) labeled goat anti-mouse IgG or anti-rabbit antibody were from Beijing Zhongshan Biotech Company (Beijing, China). Other reagents are analytical grade from commercial sources.

### Hederagenin preparation

One hundred g leaves of ivy (Hedera helix L.) were immersed in 90% ethanol (v/v) for two hours, then refluxing was used to extract three times with the 3000 mL ethanol every two hours. Extraction liquid of three times was merged and filtered. The filtrate was rotary evaporated to recycle the ethanol to obtain the crude extract and then was added to 30 times acid hydrolyzate solution, which was consisted of 95% ethanol and hydrochloric acid. The liquid volume fraction of hydrochloric in mixed liquid was 7%, while ethanol was 50%. The process of three-hour acid hydrolysis was set at the temperature of 80°C. After acid hydrolysis, the mixed liquid was recovered ethanol to without alcohol taste. The liquid was allowed to cool and deposit at room temperature. Precipitate, was washed with a little distilled water and dried under reduced pressure at 65°C to prepare hederagenin. The chemical structure of this compound has been identified.

### Cell culture

Human LoVo colon cancer cell line was obtained from Type Culture Collection, Chinese Academy of Sciences (Shanghai, China) and incubated in DMEM supplemented with 10% fetal bovine serum (FBS), penicillin (100 U/ml) and streptomycin (100 μg/ml) at 37°C in a water-saturated atmosphere with 5% CO_2_. These cells were cultured in 25 cm^2^ cell culture flasks. Medium was replaced every 2 days. Cells treated with equal amount of vehicle (dimethyl sulfoxide, DMSO) alone were used as a negative control.

### MTT assay

MTT assay was performed to determine cell viability according to the previous method [13]. Briefly, LoVo cells were seeded into 96-well plates at a density of 10,000 cells/well. After being treated with 0.25, 0.5, 1.0, 2.0 and 4.0 μM hederagenin for 24 or 48 h according to our pre-test results, the medium was discarded and treated with 20 μl MTT (5 mg/ml). After incubation for 4 h at 37°C, the 100 μl DMSO was added to each well to dissolve the resultant formazan crystals. Absorbance was detected at 490 nm by using ELx800 microplate reader (BioTek, Winooski, VT) [14].

### Hoechst 33342 nuclear staining

Apoptotic morphological alterations in the nuclear chromatin were detected by Hoechst 33342 staining. The cells at a density of 3 × 10^5^ per well were grown onto cover slips in 6-well plates. After incubation for 24 h, these cells were treated with 1 and 2 μM hederagenin for 48 h. These cells were washed with phosphate buffer saline (PBS) and stained with Hoechst 33342 for 30 min in CO_2_ incubator [15]. After rinsed three times in PBS, the cells were examined for nuclear changes (i.e., chromatin condensation and nuclear fragmentation) characteristic of apoptosis under fluorescence microscopy.

### Apoptosis assay

Induction of apoptosis was assessed by the binding of annexin V-FITC/PI to phosphotidylserine. Briefly, these cells (1 × 10^6^) were treated with 1 and 2 μM hederagenin for 48 h. Cells were harvested and suspended in binding buffer. An aliquot of 300 μl was incubated with 3 μl of Annexin V-FITC and 3 μl of propidium iodide (PI) for 20 min at room temperature in the dark. The stained cells were analyzed by fluorescent activated cell sorting (FACS) on a FACScan flow cytometer (Becton Dickinson) at an excitation wavelength of 488 nm and observation wavelengths of 530 and 575 nm [16, 17].

### Measurement of mitochondrial membrane potential

Mitochondrial membrane potential was assessed by measurement with 5,5′,6,6′-tetrachloro-1,1′,3,3′-tetraethyl-benzamidazolylcarbocyanine iodide (JC-1;Molecular Probes; T3168). Cells (1 × 10^6^) were treated with 1 and 2 μM hederagenin, harvested and washed twice with cold PBS. The cells were incubated for 30 min at 37°C with the fresh culture medium containing JC-1 of 2.5 μg/ml. Cells were collected by centrifugation at 2000 *g* for 5 min and incubated with 10 μg/ml JC-1 dye for 30 min, and then washed once with DMEM. Both red and green fluorescence emissions were analyzed by flow cytometry using an excitation wavelength of 488 nm and emission wavelengths of 530 nm (green fluorescence)/585 nm (red fluorescence). An increase in green fluorescent (FI) intensity represents mitochondrial swelling, whereas a decrease in red fluorescence indicates loss of mitochondrial membrane potential [18].

### Flow cytomertry detection of reactive oxygen species (ROS)

The levels of ROS in LoVo cells were stained by 2,7-dichlorodihydrofluorescein diacetate (DCFH-DA, Sigma) and examined by flow cytometry. The cells (1 × 10^6^ cells/ml) were treated with 1 and 2 μM hederagenin for 48 h to detect the changes of ROS. These cells were harvested and washed twice with PBS, and then re-suspended in 500 μl of DCFH-DA solution (10 μM). After being incubated at 37°C for 30 min, the levels of ROS were analyzed by flow cytometry at an excitation 488 nm and emission 525 nm.

### Quantitative real-time PCR

Total RNA was isolated by using the TRIzol reagent (Invitrogen, Carlsbad, CA) following the manufacturer’s instructions. Briefly, after being treated with hederagenin for 48 h, these cells were resuspended in 1 ml of TRIzol. The suspension was extracted with 0.2 ml of chloroform. After being centrifuged with 2000 *g* for 5 min and mixed with 0.5 ml of isopropyl alcohol, the resultant pellet was washed with 0.7 ml of 75% ethanol and finally resuspended in 50 μl RNase-free water. All total RNA samples were kept at −80°C. The primers for human cDNA synthesis were performed using iScript select cDNA synthesis kit (Bio-Rad). Each sample was tested in triplicate, with the use of the Quantitect SYBR Green PCR kit (Qiagen, Hilden, Germany) for 40 cycles (95°C for 10 min, 95°C for 15 s, and 60°C for 1 min) on the ABI 7900HT fast real time PCR System (Applied Biosystems, Foster, CA). The primers were as follows: β-actin forward primer, GGCCAACCGCGAGAAGAT, β-actin reverse primer, CGTCACCGGAGTCCATCA; Bax forward primer, TTTGCTTCAGGGTTTCATCC, Bax reverse primer, GCCACTCGGAAAAAGACCTC; Bcl-2 forward primer, ATGAACTCTTCCGGGATGG, Bcl-2 reverse primer, TGGATCCAAGGCTCTAGGTG; Bcl-xL forward primer, TCGCCCTGTGGATGACTGAG, Bcl-xL reverse primer, CAGAGTCTTCAGAGACAGCCAGGA; Survivin forward primer, TTCTCAAGGACCACCGCATC; Survivin reverse primer, GCCAAGTCTGGCTCGTTCTC. Cycle threshold (Ct) values were obtained graphically for the target genes and β-actin. The difference in Ct values between GAPDH and target genes were represented as ∆Ct values. ∆∆Ct values were obtained by subtracting ∆Ct values of control samples from those of treated samples. The relative fold change in gene expression was calculated as 2-∆∆Ct [19, 20].

### Protein extraction and western blotting analysis

These cells were treated with 1 and 2 μM hederagenin in 100 mm-diameter culture dishes for 48 h. After treatment, the cells were washed twice with ice-cold PBS, and harvested by scraping in 200 μl of lysis buffer [20 mM Tris–HCl (pH = 8.0), 1 mM sodium orthovanadate, 10% glycerol, 1 mM phenylmethylsulfonyl fluoride, 2 mM EDTA, 1% Triton X-100, 50 mM β-glycerolphosphate and 10 mg/ml each of aprotinin, leupeptin and pepstatin]. Eighty micrograms of proteins which was determined by BCA protein assay kit was separated electrophoretically using a 12% sodium dodecyl sulfatepolyacrylamide gel electrophoresis (SDS-PAGE) gel and transferred to a polyvinylidene fluoride (PVDF) membrane. The membrane was incubated at 4°C overnight in 5% skim milk in TBST (20 mM Tris–HCl, pH 7.6, 150 mM NaCl, and 0.05% Tween-20) containing primary antibodies at one of the following: Bax (1:1000), Bcl-2 (1:1000), caspase-3 (1:500), caspase-9 (1:500), PARP (1:500) or β-actin (1:1000). After washing with TBST for three times, the membrane was incubated with goat anti-rabbit IgG HRP conjugated secondary antibody (1:1000) or goat anti-mouse IgG HRP conjugated secondary antibody (1:1000) for 1 h at room temperature [21, 22]. Immunodetection was performed with an enhanced chemiluminescence (ECL) detection kit (Cell Signaling Technology, Beverly, MA). The protein brands were analyzed using densitometry scanning with the Chemilmager™ 5500 fluorescence system equipped with the analysis software AlphaEase FC™ (Alpha Innotech Corporation, San Leandro, CA 94577, USA).

### Measurement of lactate dehydrogenase

To measure lactate dehydrogenase (LDH) release, 100 μl/well supernatant medium collected from above Hoechst 33342 staining experiment was transferred to the corresponding well of an optically clear 96-well flat-bottom microtiter plate and analyzed using an LDH cytotoxicity detection kit (KeyGEN, Nanjing, China), according to the manufacturer’s instructions. LDH activity was calculated by measuring the increase in absorbance at 450 nm.

### Statistical analysis

All data are from at least three individual experiments and are expressed as mean ± standard deviation (SD). Statistical comparisons of the results were evaluated using analysis of variance (ANOVA) with SPSS 13.0. Significant differences between the control and treated cells were analyzed by Student’s *t*-test (*p < 0.05 or **p < 0.01)

## Results

### Effects of hederagenin on LoVo cell viability

**Figure 2:**
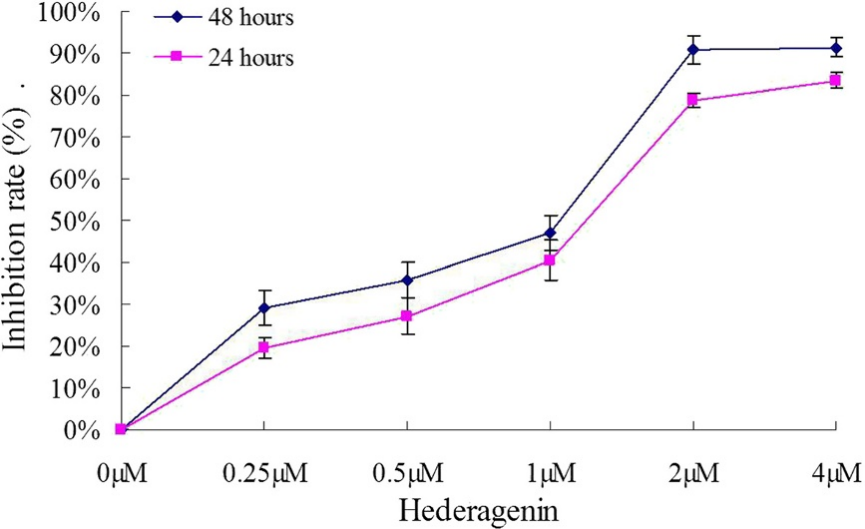
**The effect of hederagenin on cell proliferation of LoVo cells.** The cells were incubated with the indicated concentrations of hederagenin for 24 and 48 h. DMSO of 0.04% was used as the control. The data are expressed as means ± standard deviation from three independent experiments (n = 12).

The effect of hederagenin on LoVo cell viability was examined by MTT assay (Figure 2). LoVo cells were treated with hederagenin (0.25, 0.5, 1.0, 2.0 and 4.0 μM) for 24 and 48 h, respectively. The exposure of LoVo cells to hederagenin resulted in a significant decrease of cell viability. After treatment with hederagenin (1.0, 2.0 and 4.0 μM), the inhibitive rates were 40.50 ± 4.33%, 78.78 ± 5.21% and 83.44 ± 3.77% for 24 h while 47.16 ± 6.37%, 90.78 ± 5.39% and 91.35 ± 5.22% for 48 h. The IC_50_ for hederagenin in LoVo cells were 1.39 μM at 24 h for and 1.17 μM at 48 h, respectively. These results suggested that hederagenin showed a dose-dependent and time-dependent inhibitive effect on the proliferation of LoVo cells.

### Effects of hederagenin-induced LoVo cell apoptosis

**Figure 3:**
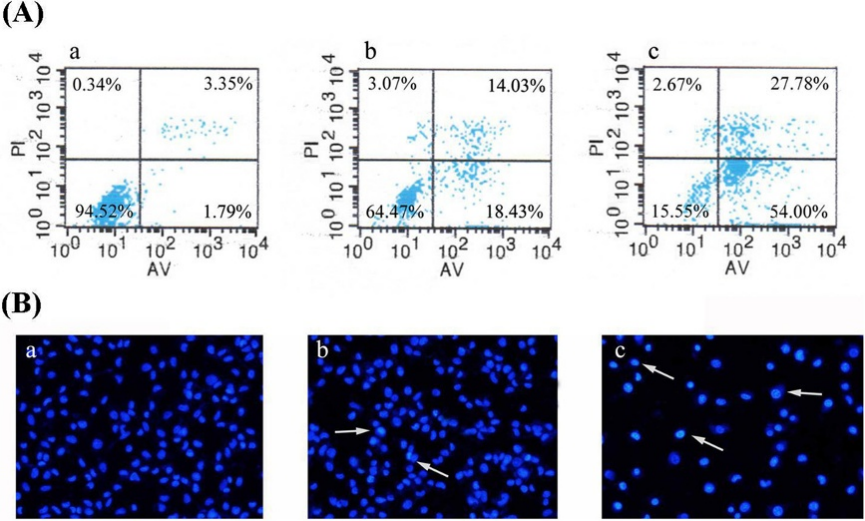
**Hederagenin-induced apoptosis (A) and apoptotic morphological changes (B) in LoVo cells.** a, control; b, 1 μΜ hederagenin; c, 2 μΜ hederagenin. LL(lower left), living cells (AV/PI negative); LR (lower right), early apoptotic cells (AV positive/PI negative); UR (upper right), late apoptotic cells (AV positive/PI positive); UL(upper left), necrotic cells(AV negative/PI positive). The numbers represent the percentage of the cells in the sum of upper right and lower right quadrants. For apoptotic morphological changes, apoptotic cells were detected by Hoechst 33342 staining and measured by fluorescence microscopy (magnification 200×).

To further confirm the apoptosis of hederagenin, LoVo cells were stained with Annexin V/PI, and subsequently analyzed by flow cytometry. As indicated in Figure 3A, after being treated for 48 h, the percentage of apoptotic cells was increased by hederagenin. The total apoptotic rates of hederagenin (1 and 2 μM) were 32.46% and 81.78%, respectively, whereas the apoptotic rate was only at 1.57% for the control. To investigate the morphological alterations after hederagenin treatment, cells were monitored using Hoechst 33342 staining. Herein, the characteristic features of apoptosis including chromatin condensation and nuclear fragmentation in hederagenin-treated cells were observed as compared with the control cells (Figure 3B). These results demonstrated that the inhibition of hederagenin on the growth of LoVo cells was associated with its induction of apoptosis.

### Effects of hederagenin on mitochondrial membrane potential disruption and ROS level

**Figure 4:**
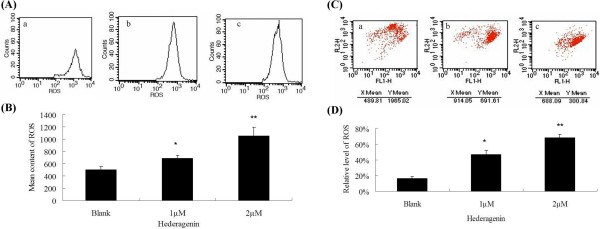
**The level of ROS after treatment were measured using DCFH-DA (A, B) or JC-1 stainings (C, D) by flow cytometry.** a, control; b, 1 μΜ hederagenin; c, 2 μΜ hederagenin. The mitochondrial membrane potential (MMP) after treatment were measured using JC-1 staining by flow cytometry. The reduced MMP indicated by a decrease in red fluorescence. The green gluorescence represents the monomeric form of JC-1. Each experiment was conducted in triplicate. *P < 0.05, **P < 0.01, vs. control.

ROS has been found to play an important role in inducing apoptosis of tumor cells. To observe the level of ROS, the LoVo cells were stained with DCFH-DA, and subsequently analyzed by flow cytometry. After treated with 1 and 2 μM hederagenin, the ROS level in LoVo cells was increased as compared with blank control (*P < 0.05, **P < 0.01). the treatment of hederagenin led to an increase of ROS levels in the examined LoVo cells (Figure 4A and B).

An involvement of mitochondria in the mechanisms of hederagenin-induced apoptosis was determined according to a ratio of red fluorescence of JC-1 aggregates and green fluorescence of JC-1 monomers. The aggregation of monomers is directly correlated to mitochondrial membrane potential, and their breakdown in dying cells results in a increase of green fluorescence. As shown in Figure 4C and D, JC-1 aggregates accumulated in control cells and displayed high ratio of red fluorescence. Hederagenin (1 and 2 μM)-treated cells resulted in a higher green fluorescence with a decrease of red fluorescence intensity. The decrease of red/green fluorescence ratio showed that the disruption of MMP were 53.81 ± 5.15% and 31.41 ± 4.23%, respectively (*P < 0.05, **P < 0.01). Data showed that hederagenin could down-regulate MMP, indicating the important role of mitochondria in hederagenin-induced apoptosis.

### Effects of hederagenin on mRNA expression levels of Bax, Bcl-2, Bcl-xL and Survivin

**Figure 5:**
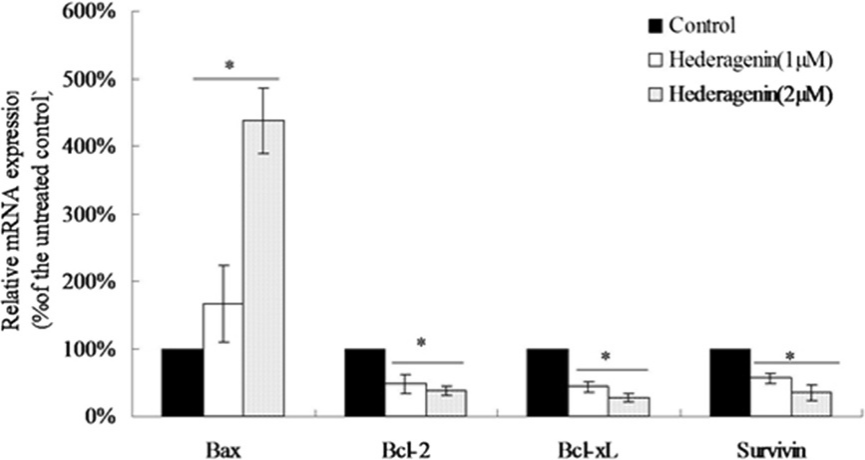
**Effects of hederagenin on mRNA levels of Bcl-2, Bcl-xL, Bax and Survivin.** The mRNA expressions were measured by quantitative RT-PCR and calculated as % of DMOS-only control. β-actin was used as an internal control. *P < 0.05, vs. control (n = 3).

Bcl-2 family was served as vital regulators of the mitochondrial pathway involved in apoptosis. To investigate whether hederagenin could regulate the Bcl-2 family including Bax, Bcl-2, Bcl-xL, and Survivin mRNA, RT-PCR was used to quantity the levels of these mRNA. Figure 5 showed that hederagenin at the concentration of 1 and 2 μM could increase Bax mRNA level while decrease Bcl-2, Bcl-xL and Survivin mRNA level in LoVo cells. These results indicated that the induction of hederagenin on apoptosis might be associated with Bax, Bcl-2, Bcl-xL and Survivin.

### Effects of hederagenin on the expression of Bax, Bcl-2 and caspases

**Figure 6:**
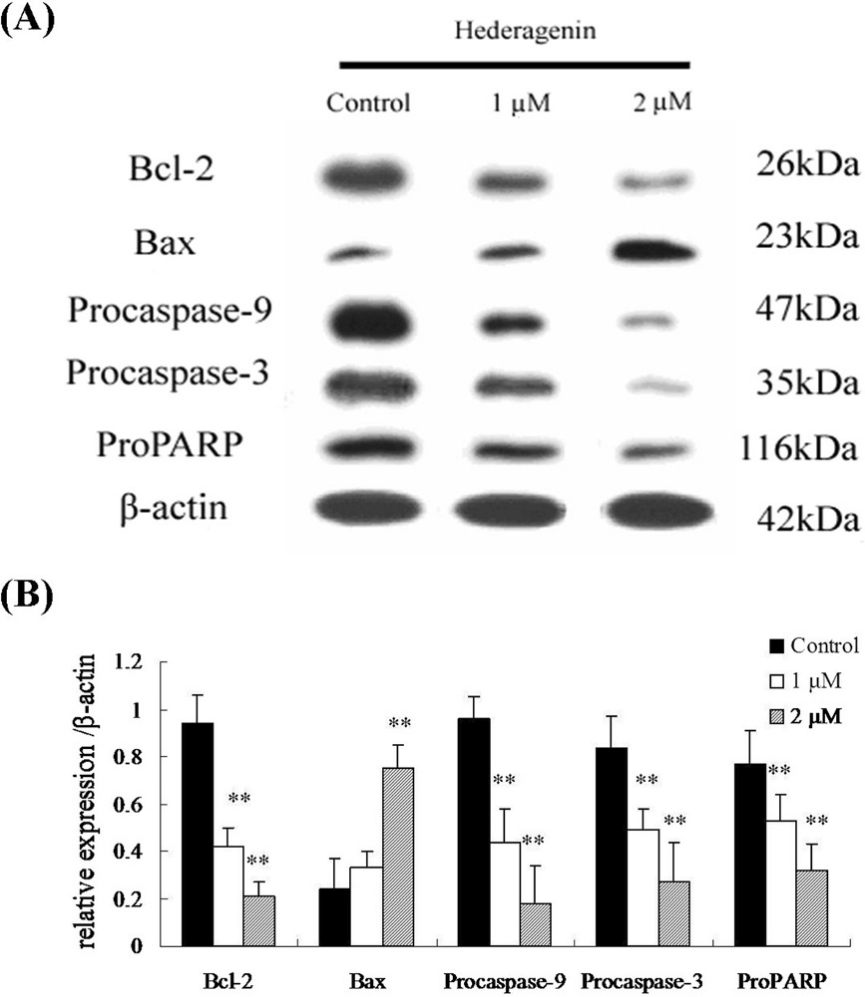
**The effects of hederagenin on the expressions of apoptosis-related proteins (A) and their protein levels (B), such as Bax, Bcl-2, procaspase-9, procaspase-3 and proPARP in LoVo cells.** Expressions of Bcl-2, procaspase-9, procaspase-3, proPARP were significantly reduced when the LoVo cells were treated with 1 and 2 μΜ hederagenin for 48 h, while the reverse was true for the expression of Bax. The data are expressed as means ± standard deviation from three independent experiments (n = 3).

**Figure 7:**
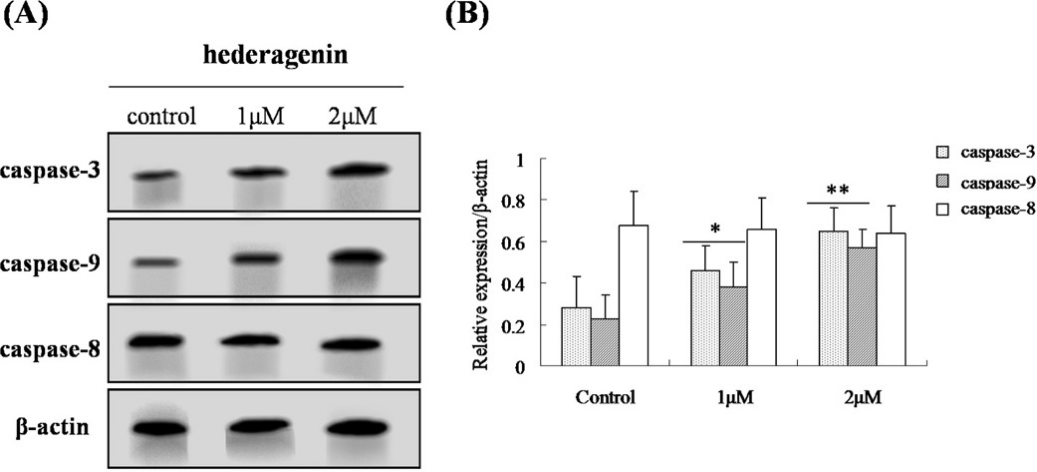
**The effects of hederagenin on the expressions of caspase-9, caspase-3 and caspase-8 (A) and their protein levels (B) in LoVo cells.** The data are expressed as means ± standard deviation from three independent experiments (n = 3).

To explore the regulation of hederagenin on apoptotic proteins, western blot analysis was conducted to evaluate the expressions of Bcl-2, Bax, procaspase-9, procaspase-3 and proPARP proteins. After treatment with hederagenin for 48 h, a significant decrease of Bcl-2 (26 kDa), procaspase-9 (47 kDa), procaspase-3 (35 kDa), proPARP (116 kDa) in LoVo cells was observed in 1 or 2 μM hederagenin-treated LoVo cells groups compared with control group (*P* < 0.01). However, the protein expression of Bax was (23 kDa) significantly increased by hederagenin (1 or 2 μM) (Figure 6A and B). The results of western blotting analysis demonstrated that the inducing apoptosis of hederagenin in LoVo cells through modulating Bcl and caspase families pathway, which was associated with mitochondrial membrane potential disruption. In order to confirm this pathway, caspase-9, caspase-3 and caspase-8 activation were observed in LoVo colon cancer cells. As shown in Figure 7A and B, hesderagenin increased caspase-9 and caspase-3 activation. However, there were no significant changes on caspase-8 activation. The resutls indicated that the induction of hederagenin on apoptosis of LoVo colon cancer cells might be achieved via intrinsic pathway rather than extrinsic pathway.

### LDH activity of hederagenin on LoVo cell

**Figure 8:**
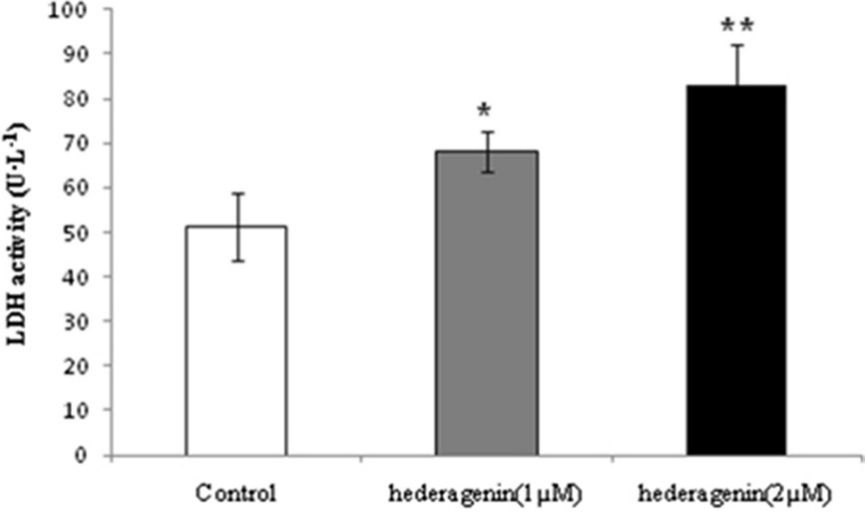
**The effects of hederagenin on the activities of LDH in LoVo cells.** The data are expressed as means ± standard deviation from three independent experiments (n = 3). *P < 0.05, **P < 0.01, vs. control.

LDH activity in the medium treated with hederagenin was significantly increased (*P < 0.05, **P < 0.01) (Figure 8). The result showed that the activities of LDH when treated with hederagenin (1 or 2 μM) were 68.25 ± 4.63 and 82.96 ± 9.11 U/L, respectively.

## Discussion

It has also been shown that hederagenin isolated from the leaves of ivy (Hedera helix L.) is effective in decreasing cell numbers in the human LoVo colon cancer cell line through inbibiting proliferation and inducing apoptosis. Briefly, apoptosis is mediated by two central pathways: the extrinsic (or death receptor) pathway and the intrinsic (or mitochondrial) pathway [23]. The extrinsic pathway is associated with Fas death receptor-mediated caspase-8 activation and then initiates the activity of downstream caspase-3 [23–25]. Apoptosome formation leads to the activation of executioner caspase-3, −6 and −7 [26]. The release of cytochrome C, which occurred at the same time as the processing of caspase-3, −8 and −9, has been regarded as a preceding event for the activation of caspase cascades [27].

The lipophilic cationic probe JC-1 was used to estimate the release of cytochrome C and demonstrate the disruption of mitochondrial membrane potential (MMP). ROS are byproducts of normal cellular oxidative processes and generated in and around mitochondria. It has been indicated that they can trigger cytochrome C release, caspase activation and subsequent apoptotic biochemichal changes [28]. In this study, DCFH-DA was also used to determine ROS levels. Our results indicated that the induction of hederagenin on apoptosis in LoVo cells might be related with the mitochondrial membrane potential disruption.

Bcl-2 family play an important role in cell apoptosis [29, 30]. Multidomain and BH3-only proapoptotic Bcl-2 proteins are antagonized by antiapoptotic family members, including Bcl-2 and Bcl-xL [31, 32]. It was observed that Bcl-2 or Bcl-xL promote cell survival by preserving the integrity of the external mitochondrial membrane and prevented the release of cytochromec from mitochondria [33]. Survivin exists in a novel mitochondrial pool in turmor cells [34]. In response to cell death stimulation, mitochondrial survivn is rapidly discharged in the cytosol to prevent caspase-3 activation and to inhibit apoptosis [35, 36]. We examined the level of Bcl-2, Bcl-xL and Survivin, as well as Bax in LoVo cells after treatment with different concentrations of hederagenin by real-time PCR or western blot. It was observed that hederagenin induced the up-regulation of Bax and down-regulation of Bcl-2, Bcl-xL and Survivin.

Caspases, a family of cysteine acid proteases, are known to act as important mediators of apoptosis and contribute to the overall apoptotic morphology by the cleavage of various cellular substrates [37]. Caspases activation resulted from cytochrome c release can stimulate mitochondrial permeability transition (MPT) [38]. The intrinsic pathway of apoptosis is associated with the activation of caspase-9, which cleaves and activates caspase-3 [39]. In this study, it has been demonstrated that the treatment of hederagenin increased the expressions of procaspase-9, procaspase-3 and proPARP. Furthermore, we also observed that hesderagenin increased caspase-9 and caspase-3 activation. However, no significant changes on caspase-8 were observed in LoVo colon cancer cells. It suggested that hederagenin induced apoptosis of LoVo colon cancer cells via intrinsic pathway rather than extrinsic pathway.

Lactate dehydrogenase (LDH) is a glycolytic enzyme. It is presented in all tissues of the body within the cytoplasm. Once the cells are damaged, LDH was then released into extracellular, the extent of cell damage can be detected by LDH activity [40]. The higher the LDH activity, the more serious the cell injured [41]. We observed that LDH activity was significantly increased by hederagenin, and the dose of 2 μM could increase LDH activity more obviously than the group with 1 μM. This indicates that hederagenin has the effect on injury LoVo colon cancer cells.

In the previous report of Park [42], hederagenin which was isolated from stem bark of Kalopanax pictus Nakai (Araliaceae) exhibited protruding cytotoxicity to some extent on several types of tumor cells and mice bearing Colon 26 and 3LL Lewis lung carcinoma. Also, some research showed that MB, a hederagenin saponin extracted from Lonicera macranthoides, exhibited strong anti-tumor effect and mitochondrion-mediated apoptosis induction involved in it [11]. Our results provide more comprehensive supplement for the mechhanism of hederagenin on colon cancer. More research should be conducted to reveal the underlying mechanism of hederagenin on inducing apoptosis of colon cancer cells.

## Conclusions

In conclusion, we demonstrated for the first time that hederagenin, isolated from the leaves of ivy (Hedera helix L.), could induce apoptosis of LoVo cells through the mitochondrial apoptotic pathway. Hederagenin could induce the up-regulation of Bax and down-regulation of Bcl-2, Bcl-xL and Survivin, the release of cytochrome c, followed by procaspase-9, procaspase-3 and proPARP cleavage. Our finding indicated that hederagenin might be a promising therapeutic candidate for the prevention and treatment of human colon cancer.
